# Electromyographic Analysis of Shoulder Neuromuscular Activity in Women Following Breast Cancer Treatment: A Cross-Sectional Descriptive Study

**DOI:** 10.3390/jcm9061804

**Published:** 2020-06-10

**Authors:** Virginia Prieto-Gómez, Beatriz Navarro-Brazález, Óscar Sánchez-Méndez, Pedro de-la-Villa, Beatriz Sánchez-Sánchez, María Torres-Lacomba

**Affiliations:** Physiotherapy in Women’s Health (FPSM) Research Group, Physiotherapy Department, Faculty of Medicine and Health Sciences, University of Alcalá, Alcalá de Henares, 28805 Madrid, Spain; v.prieto@uah.es (V.P.-G.); b.navarro@uah.es (B.N.-B.); osmfisio@gmail.com (Ó.S.-M.); pedro.villa@uah.es (P.d.-l.-V.); maria.torres@uah.es (M.T.-L.)

**Keywords:** persistent pain, breast cancer, shoulder motion, neuromuscular activity, electromyography

## Abstract

Certain secondary effects derived from medical treatment in breast cancer can favor the development of alterations in shoulder biomechanics. To the best of our knowledge, persistent peripheral pain as a key factor for the development of neuromuscular activity impairments has not been analyzed. A cross-sectional descriptive study was carried out. A total of 90 women were included and allocated to three groups: (i) 30 women with persistent peripheral pain after breast cancer treatment, (ii) 30 women without pain after breast cancer treatment, and (iii) 30 healthy women. Surface electromyography was employed to measure the onset and amplitude of the muscle activity of three shoulder movements. Statistically significant differences were found in the neuromuscular activity for all the muscles and shoulder movements among women with persistent pain versus healthy women (i.e., amplitude muscle activity variable *p* < 0.001). Statistically significant differences were also observed in the neuromuscular activity for certain muscles in shoulder movements among women with persistent pain versus women without pain, as well as between women without pain versus healthy women. Therefore, following breast cancer treatment, women showed alterations in their shoulder neuromuscular activity, which were more significant if persistent pain existed. These findings may contribute to developing a selective therapeutic exercise program that optimizes the shoulder neuromuscular activity in women after breast cancer treatment.

## 1. Introduction

The improvement in the effectiveness of breast cancer medical treatment has increased the survival rate to between 78%–88% at five years [1]. Unfortunately, many women continue to experience consequences following breast cancer treatment, including persistent pain and disability, tissue tightness [2,3,4,5,6,7,8,9], and decreases in the health-related quality of life [1]. Anterior chest or axillary tissue alterations [9], myofascial pain syndrome [9], heightened mechanosensitivity of neural tissue [10], and shoulder symptoms could result in altered shoulder biomechanics [2,3,5,6,7,8,11,12]. Shoulder movement during elevation requires a correct synchronization between the scapula, the clavicle, and the humerus, so altered movement in any segment could impact the others, increasing the risk of shoulder dysfunction [13].

For instance, during arm elevation, the normal movement of the scapula consists of upward rotation, posterior tilt, and internal rotation, and many studies related the shoulder pain to a decrease in the upward rotation and posterior tilting of the scapula, and an increase in the scapular internal rotation [14,15,16,17,18]. Scapular kinematic alterations are commonly found in breast cancer survivors [2,3,5,7,8,19,20]. Suitable muscle activity in timing and amplitude is needed for optimal movement [21,22,23]. Similarly, alterations in shoulder neuromuscular activity were found in women following breast cancer treatment. These alterations depend on the type of surgery, operated side and breast cancer treatment received [2,5,8,11,20].

The influences of persistent pain on these motor changes should be studied. Neuromuscular activity alterations in women following breast cancer treatment could be linked to neuroplasticity changes in the central nervous system [24,25,26,27]. These changes in neuromuscular activity were observed in shoulder dysfunctions [14,15,16,17]. In addition, these neuroplasticity changes were associated with a poor prognosis, resulting from conventional treatments [27]. Regarding this, determining whether alterations occur in the shoulder neuromuscular activity is important for deciding to apply a more contemporary and therefore more effective physiotherapy treatment, and offering the possibility of a preventive approach [24,27]. Thus, we aimed to describe differences in shoulder neuromuscular activity among women with peripheral persistent pain after breast cancer treatment versus women without pain after breast cancer treatment versus healthy women; and whether differences exist among women without pain after breast cancer treatment versus healthy women.

## 2. Experimental Section

### 2.1. Design

A cross-sectional descriptive study was performed between January 2015 and May 2019 at the Research Unit of the “Physiotherapy in Women’s Health Research Group” of the University of Alcalá (Madrid, Spain). The study was approved by the Human Research Ethics Committee at the University of Alcalá, Madrid, Spain (protocol number: 2013/014/20130624).

### 2.2. Participants

The sample were enrolled from the Research Unit of the “Physiotherapy in Women’s Health Research Group” of the University of Alcalá (Madrid, Spain). 

Women who fulfilled the inclusion criteria were informed about the study and invited to participate. In the case of agreement, they freely signed the informed consent prior to participation.

The women included were allocated to one of the three groups: group 1, group 2, or group 3.

Group 1 (G1): The inclusion criteria were: eligible women with persistent pain after breast cancer treatment (surgery and/or radiotherapy and/or chemotherapy) in the dominant side with pain for at least 6 months, aged between 45 and 65 years old (inclusive). Women receiving hormone therapy during the study were also included. The established exclusion criteria were: shoulder pain episodes in the dominant side prior to breast cancer treatment, bilateral breast cancer, predominant central sensitization pain identified using the Central Sensitization Inventory (>40 points) [28], vascular conditions following breast cancer treatment, infection, fever, metastasis and locoregional recurrence, neurological disorders, intake of analgesics, anti-inflammatories, or medications that could alter the response of the nervous system, shoulder forward flexion and abduction range of motion inferior to 90 degrees, and cognitive limitations to understanding the information (taken from medical report), instructions, and consent for their participation in the study.

Group 2 (G2): Selection criteria for G2 were the same to G1, but in the G2, only eligible women without persistent pain after breast cancer treatment were included.

Group 3 (G3): The inclusion criteria were: healthy women, aged between 45 and 65 years old (inclusive). The established exclusion criteria were: shoulder pain episodes in the dominant side; neurological disorders; intake of analgesics, anti-inflammatories, or medications that could alter the response of the nervous system; joint range of shoulder forward flexion and abduction range of motion inferior to 90 degrees; and cognitive limitations to understanding the information (taken from medical report), instructions and consent for their participation in the study.

### 2.3. Sample Size Estimation

With a sample size of 30 participants in each arm, we used 80% power to detect a difference of 0.67 s in the onset of the serratus anterior muscle activity during the shoulder abduction movement between G1 versus G3, assuming a standard deviation of the change of 0.92 s with a significance level of α = 0.05. With a sample size of 30 participants in each arm, we used 80% power to detect a difference of 7.2% in the amplitude of the serratus anterior muscle activity during the shoulder abduction movement between G1 versus G3, assuming a standard deviation of the change of 9.87% with a significance level of α = 0.05. The serratus anterior muscle was selected as one of the main stabilizing muscles of the inferior angle and the medial border of the scapula [14,15,17], as well as one of the muscles most affected by the medical treatment for breast cancer [3,7,9]. The calculation of the sample size was conducted using Granmo version 7.12 software (URLEC-IMIN, Barcelona, Spain).

### 2.4. Data Collection

#### 2.4.1. Shoulder Neuromuscular Activity

Surface electromyography (sEMG) was used to measure the onset and amplitude of muscle activity for five different muscles of the shoulder, performing the three movements that are more affected in women after breast cancer (abduction, forward flexion, and external rotation) [29]. In all the movements that were evaluated, the upper trapezius muscle, lower trapezius muscle, and especially the serratus anterior muscle was established as representatives for the upward scapular rotator muscles. The infraspinatus muscle was chosen to represent the rotator cuff muscle group [30]. The middle deltoid muscle was selected as the representative for shoulder abduction, due to it being a prime mover in this movement [30]. Due to their high relevance in the participation during the execution of their respective movements, the anterior deltoid muscle [31] was selected for shoulder forward flexion movement, and the posterior deltoid muscle [32] for shoulder external rotation movement.

**Figure 1:**
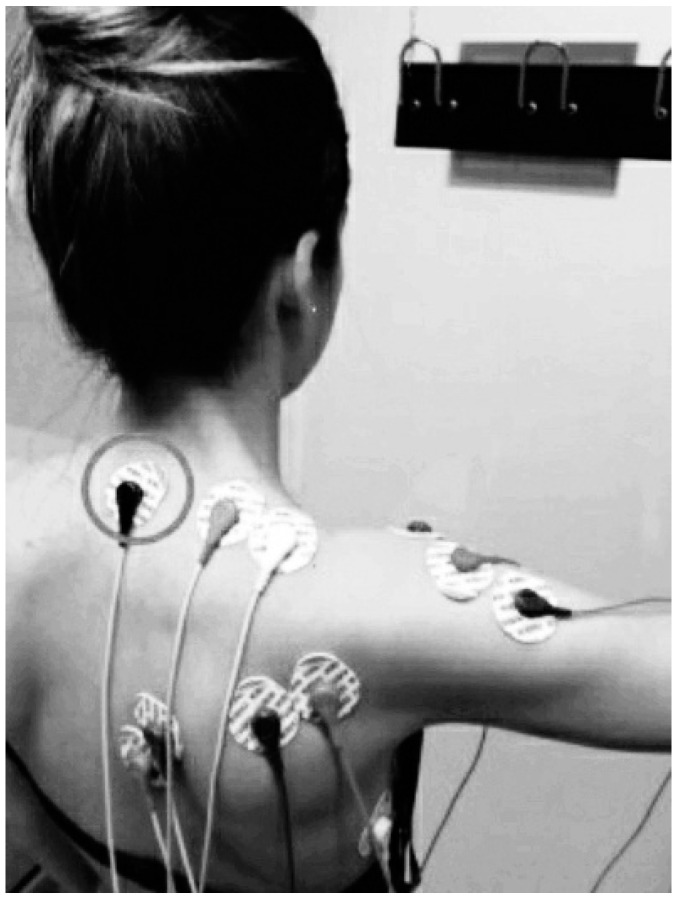
Posterior view of electromyography electrodes position during isometric shoulder abduction contraction. The reference electrodes are indicated with a circle. Image of Prieto Gómez reproduced with permission of the participant; 2019.

**Figure 2:**
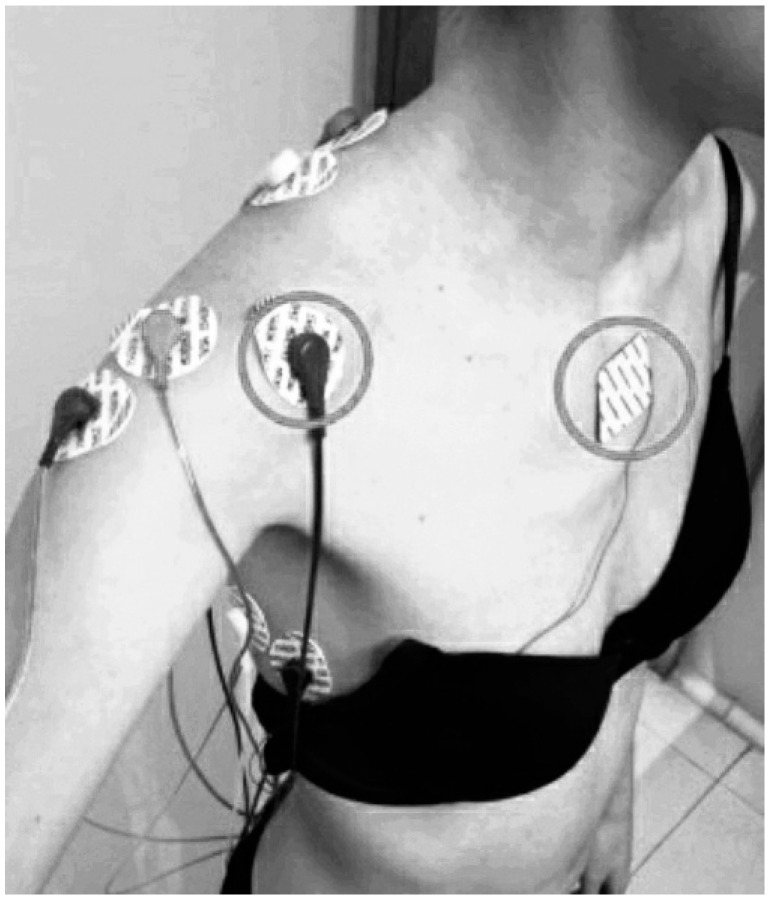
Anterior view of electromyography electrodes position during isometric shoulder abduction contraction. The reference electrodes are indicated with a circle. Image of Prieto Gómez reproduced with permission of the participant; 2019.

Bipolar Ag/AgCl electrodes (KendallTM 100 Series Foram Electrodes, Covidien, MA, USA) were positioned in accordance with Cram andKasman [30,33]. sEMG data were captured with a five-channel data recording system 16-bit data acquisition A/D board PowerLab 8/30 (ADInstruments, Sydney, Australia), and processed with LabChart v.7 software (ADInstruments, Sydney, Australia). The sEMG signal was amplified with a gain of 1000, filtered (10–500 Hz), rectified, and smoothed, and the root mean square (RMS) was quantified. The sampling frequency was 1000 Hz [30,34]. The electromyographic signal for the infraspinatus muscle was amplified by 10,000 using an external amplifier (Amplifier AC, CP511, Ast-Med, Inc. Grass Product Group, Warwick, NY, USA). (Figure 1 and Figure 2).

##### The Onset of Muscle Activity

Every woman performed these movements three times with a 5-s rest between attempts to re-establish a baseline signal:

(1) Shoulder abduction movement: Elevation of the arm in the scapular plane from rest position, until 90 degrees was reached.
(2) Shoulder forward flexion movement: Elevation of the arm in the sagittal plane of motion from rest position, until 90 degrees was reached.
(3) Shoulder external rotation movement: External movement (outward) turning around the vertical axis in the transverse plane of motion with the arm at 90 degrees of elevation in the scapular plane and the elbow in the flexion position. Movement from 0 degrees until 30 degrees was reached.

To obtain the onset of muscle activity, the algorithm proposed by Hodges and Bui was used. For a low-noise signal analysis: 10 ms windows, one standard deviation respect to the baseline signal with a 500 Hz low pass filter [30]. To detect the onset of movement, a Microsoft^®^ Kinect Spatial Vision Camera synchronized with the electromyographic equipment was used [35,36]. Muscles that were activated before the movement took negative values less than 0.

##### The Amplitude of Muscle Activity

Women performed three trials of the movement with a 5-s rest between the trials to re-establish a baseline signal:

(1) Shoulder abduction movement: A 5-s isometric contraction at 90 degrees of elevation of the arm in the scapular plane.
(2) Shoulder forward flexion movement: A 5-s isometric contraction at 90 degrees of arm elevation in the sagittal plane of motion.
(3) Shoulder external rotation movement: A 5-s isometric contraction at 0 degrees of external movement (outward) turning around the vertical axis in the transverse plane of motion, with the arm at 90 degrees of elevation in the scapular plane and the elbow in the flexion position. Prior to isometric contraction, women maintained that arm elevation and flexion elbow position during 5-s to re-establish a baseline signal.

The average of RMS of 500 ms was calculated in all muscles [33,34]. The values were normalized based on the difference in activation amplitude with respect to the baseline RMS (RMS%) (500 ms) [5,8,20].

Women were instructed to stop the tasks if pain was experienced.

#### 2.4.2. Other Outcomes

Demographic and anthropometric data were collected: age, body mass index (BMI), education level, and occupation. Breast cancer therapies, the vascular condition following breast cancer treatment, pain intensity, and pain characteristics were also registered in G1.

### 2.5. Data Analysis

The quantitative variables were described by means and standard deviations, and the categorical variables were described with absolute values and frequencies. The assumption of normality was verified by the Shapiro–Wilk statistical test and the histogram graphs. One-way ANOVA was used as a hypothesis test and the Bonferroni test was used for multiple comparisons. IBM SPSS version 24.0 software (IBM, Armonk, NY, USA) was used for the statistical analysis.

## 3. Results

**Figure 3:**
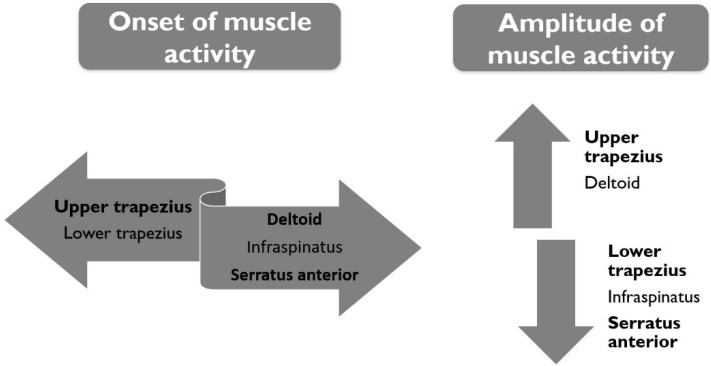
Summary data of shoulder muscle activity in women after breast cancer treatment.

A total of 90 women participated in this transversal descriptive study. Data on the demographic, anthropometric, therapeutic, and clinical variables of women treated for breast cancer and the demographic and anthropometric variables related to healthy women are presented in Table 1. Women pain characteristics were classified following guidelines for the clinical classification of predominant neuropathic, nociceptive and central sensitization pain according by Nijs et al. [37].The summary data for the shoulder muscle activity in women after breast cancer treatment are shown in Figure 3.

### 3.1. The Onset of Muscle Activity

#### 3.1.1. Shoulder Abduction Movement

Statistically significant differences were found in the onset of the lower trapezius muscle activity between G1 versus G2 (*p* = 0.001), G1 versus G3 (*p* < 0.001), and G2 versus G3 (*p* < 0.001). We also found early muscle activation on both G1 and G2 compared to G3 as well as G1 versus G2. Similarly, statistically significant differences were found for all of the groups in the middle deltoid muscle (G1 versus G2: *p* < 0.001; G1 versus G3: *p* < 0.001; G2 versus G3: *p* = 0.004), showing delayed activation in G1 compared with G3 and G1 versus G2. The upper trapezius muscle was activated early in G1 compared with G2 (*p* = 0.004) and G3 (*p* = 0.005). A delayed activation was observed in the serratus anterior muscle of G1 compared with G2 and G3 (*p* < 0.001). Finally, statistically significant differences were found in the onset infraspinatus muscle activity in relation to G1 versus G3 (*p* = 0.002) and G2 versus G3 (*p* = 0.006), showing a delayed activation in the onset of muscle activity compared to G3 (G1: 1.01 (1.81); G2: 0.90 (0.27); G3: −0.10 (1.13)) (Table 2).

#### 3.1.2. Shoulder Forward Flexion Movement

Statistically significant differences were found in the onset of the serratus anterior muscle activity between G1 versus G2 (*p* < 0.001), G1 versus G3 (*p* < 0.001), and G2 versus G3 (*p* = 0.026), finding a delay in the onset of muscle activity of G1 and G2 with respect to G3 as well as G1 versus G2. The upper trapezius muscle was activated early in G1 compared to G2 and G3 (−0.1 (0.14)) (*p* < 0.001). Similarly, the anticipated activation of the lower trapezius muscle on G1 was observed compared to G2 and G3 (*p* < 0.001). Statistically significant differences were found in the onset of infraspinatus muscle activity between G1 versus G2 (*p* = 0.028) and G1 versus G3 (*p* < 0.001), showing an anticipatory activation with respect to G3. Finally, we observed delayed activation of the anterior deltoid muscle of G1 in relation to G2 and G3 (*p* < 0.001) (Table 3).

#### 3.1.3. Shoulder External Rotation Movement

Statistically significant differences were found in the onset of activity of the upper trapezius muscle between G1 versus G3 (*p* = 0.003), showing an anticipatory activation. A delay in the onset of muscle activity of the posterior deltoid muscle was observed in G1 compared with G3 (*p* = 0.002) as well as the infraspinatus muscle when comparing G1 versus G3 (*p* = 0.002). Statistically significant differences were found in the lower trapezius muscle for G1 versus G2 (*p* = 0.006) and G1 versus G3 (*p* = 0.001), showing a delay in the muscle activation on G1 compared to G2 and G3. Finally, we observed a delay in the onset of muscle activity of the serratus anterior muscle of G1 in relation to G2 (*p* = 0.006) and G3 (*p* < 0.001) (Table 4).

### 3.2. The Amplitude of Muscle Activity (RMS%)

#### 3.2.1. Shoulder Abduction Movement

Statistically significant differences were found in relation to the RMS% of the middle deltoid muscle between G1 versus G2 (*p* = 0.001), G1 versus G3 (*p* < 0.001), and G2 versus G3 (*p* = 0.043), finding the amplitude of muscle activity increased in both G1 and in G2 compared to G3, as well as G1 versus G2. Similarly, statistically significant differences were found between all groups in the serratus anterior muscle (*p* < 0.001), with a decrease in the amplitude of muscle activity in the G1 and G2 compared with G3 and G1 versus G2. An increase in amplitude was observed in the upper trapezius muscle in G1 compared to G2 and G3 (*p* < 0.001). The infraspinatus muscle showed a decrease in the RMS% in G1 versus G2 and G1 versus G3 (*p* < 0.001). Finally, we observed a decrease in the amplitude of muscle activity in the lower trapezius muscle on G1 versus G3 (*p* < 0.001) and G2 versus G3 (*p* = 0.007) (Table 5).

#### 3.2.2. Shoulder Forward Flexion Movement

Statistically significant differences were found in relation to the RMS% of the lower trapezius muscle between G1 versus G2 (*p* = 0.020), G1 versus G3 (*p* < 0.001), and G2 versus G3 (*p* < 0.001). We found a decreased amplitude of muscle activity in G1 and G2 compared to G3, as well as in G1 versus G2. Statistically significant differences were found between all the groups in the infraspinatus and in the anterior serratus muscle (*p* < 0.001), observing a decrease in the amplitude of muscle activity in G1 versus G2, G1 versus G3, and G2 versus G3. We observed an increase in the amplitude of the anterior deltoid muscle activity on G1 versus G2 and in G1 versus G3 (*p* < 0.001). Finally, in the upper trapezius muscle, an increased amplitude of muscle activity on G1 versus G2 and G1 versus G3 was observed (*p* < 0.001) (Table 6).

#### 3.2.3. Shoulder External Rotation Movement

Statistically significant differences were found in relation to the RMS% of the lower trapezius muscle and the posterior deltoid between G1 versus G2 and G1 versus G3 (*p* < 0.001), finding a decrease in the amplitude of the muscle activity. Statistically significant differences were found in all groups (*p* < 0.001) in the infraspinatus muscle and in the serratus anterior muscle, showing a decrease in the amplitude of the muscle activity. Finally, the upper trapezius muscle increased the amplitude of the muscle activity on G1 versus G2 and G1 versus G3 (*p* < 0.001; Table 7).

## 4. Discussion

To the best our knowledge, this is the first study related to shoulder neuromuscular activity analysis in women after breast cancer treatment that considered the presence or absence of persistent pain as a key factor for the development of neuromuscular activity impairments [2,5,8,11,20].

In the present study, shoulder neuromuscular activity values, quantified by sEMG, showed alterations in the amplitude of muscle activity and the onset in each of the selected shoulder movements among the women after breast cancer treatment (G1 and G2). In this sense, these alterations were of greater magnitude if persistent pain existed. In relation to the onset of muscle activity, the upper trapezius muscle was activated early in women treated for breast cancer in all movements. This anticipation could be a compensation attempt by the upper trapezius muscle to replace the activation delay of the muscles related to the rotator cuff. If the onset of muscle activity of this set of muscles is delayed, the humeral head in the glenoid cavity does not stabilize in proper time, causing a reduction in subacromial space [12,17,18,23]. According to this, the upper trapezius muscle elevates the clavicle [17,18] to produce a wider space during the execution of the movement. 

The onset of muscle activity of the lower trapezius muscle was anticipated during the shoulder abduction and forward flexion movement in women treated for breast cancer. This is possibly due to a compensatory mechanism [24] linked to the activation delay of the serratus anterior muscle. In addition, the lower trapezius muscle and the serratus anterior muscle delayed their participation in the shoulder external rotation movement among women after breast cancer treatment. It is possible that due to the position of the shoulder during the external rotation movement (i.e., shoulder abduction at 90 degrees), places a greater demand on the neuromusculoskeletal system.

The onset of muscle activity in the anterior deltoid muscle, the middle deltoid muscle, and the posterior deltoid muscle were delayed in every shoulder movement that we evaluated. The reason could be related to the ineffective stabilization of the humeral head in the glenoid cavity by the rotator cuff [17,18,23], hindering the mobilizing action of the deltoid muscle with respect to the humerus.

The amplitude of muscle activity of the upper trapezius muscle increased markedly in women treated for breast cancer (G1 and G2) for all movements evaluated. This mechanism was also observed in different shoulder dysfunctions, including subacromial impingement syndrome [17,18,38,39]. These changes could be mediated by the central nervous system as an adaptation [24,27,40,41] to the deficiencies of the rotator cuff muscles. 

The serratus anterior muscle decreased its activity in all movements in women treated for breast cancer. This muscle has a high percentage of phasic fibers that could favor muscle inhibition [42]. Medical treatment received by women with breast cancer directly affects the anterior serratus muscle. In this sense, its alteration is usually related to the neural tissue damage of the long thoracic nerve during surgery [3,7,9], whose alteration in mechanosensitivity can be amplified and maintained by the effects of radiotherapy and chemotherapy [9,10], the presence of fibrosis and scarring [3,7,9], as well as the existence of myofascial pain syndrome [9]. Nevertheless, different studies in women following breast cancer treatment found that the amplitudes of activity of the upper trapezius muscle and serratus anterior muscle vary depending on the operated side, surgery type, and coadjutant treatment [5,8,20].

The lower trapezius muscle showed decreased activity in women treated for breast cancer, coinciding with other studies related to alterations in painful shoulders [17,18,23,39]. In the present study, the lower trapezius muscle was activated earlier; however, this did not imply an increase in the neuromuscular activity. Regarding this phenomenon, a plausible explanation may be related to the high percentage of phasic fibers in this muscle, whose presence appears to be related to muscle inhibition [42].

The amplitude of the anterior deltoid muscle and middle deltoid muscle activity increased significantly in all the movements evaluated in women treated for breast cancer, possibly to counteract the deficiencies of the rotator cuff muscles [17,18,23]. However, the posterior deltoid muscle decreased its amplitude of muscle activity. The most probable cause was that the shoulder rotation movement was conducted with a previous shoulder abduction that inhibited the action of the deltoid muscle.

Shoulder neuromuscular activity alterations are closely related to the possible appearance of changes in the scapular kinematics. In relation to the findings found in this study, an inadequate performance of the serratus anterior muscle, together with the middle trapezius muscle, is associated with the appearance of excessive scapular internal rotation [3,7]. Similarly, a decrease in the involvement of the serratus anterior muscle and lower trapezius muscle, as well as an increase in the activity of the upper trapezius muscle, favors the presence of an excessive scapular anterior tilt and internal rotation, as well as an insufficient scapular upward rotation [17,18].

In shoulder pain disorders, an increase in the neuromuscular activity of the pectorales minor [43], which could also facilitate an excessive scapular anterior tilt, was observed. Complex kinematics distortions are commonly observed in shoulder dysfunctions [18,24,44,45,46] and were reported in women after breast cancer treatment [2,5,7,19,47].

Statistically significant differences in all muscles and movements, both in the onset and amplitude of muscle activity, were found between the women with peripheral persistent pain and healthy women. In other studies, the presence of pain and movement disorders was also observed in women treated for breast cancer in the shoulder region [5,8,20,47], as well as in the cervical region [48], with painful shoulders [17,18,43,44,45], and other areas of the body such as the cervical [49], and low back [50] regions, in the general population.

The current knowledge regarding the neurophysiology of pain sheds light on the possible mechanisms involved in these neuromuscular activity changes [24,25,26,27]. Musculoskeletal tissue damage, in addition to causing damage in the anatomical structures, could provoke functional neuroplastic changes of the nervous system [24,25,26,27].

The tissue damage caused by breast cancer treatments in both myofascial tissue [9] and neural tissue [10], the adoption of defensive postures especially in acute phases [9], and the appearance of possible vascular consequences [51,52], would give rise to nociceptive stimuli that could directly trigger the activation of neurons in the somatosensory cortex, which could induce a pain-dependent inhibitory input mechanism in the primary motor cortex [24]. The cortical reorganization in this area could produce the central nervous system to create compensatory muscle activation strategies, which in turn would imply overload in the involved areas, further increasing the nociception [24]. This nociception could be further amplified by a process of peripheral [9,10,24] and/or central sensitization [9,10,24], as well as by an increase in sympathetic activity [24] related to the high levels of emotional stress and anxiety [24], which are usually present in these women [1].

In recent years, moderate evidence suggests that persistent musculoskeletal pain requires the integration of pain neuroscience education with cognition-targeted therapeutic exercises, including neuromuscular training, to alter pain memories in these patients [53]. Regarding neuromuscular training, findings in this study concerning the timing and amplitude of shoulder muscle activity could help with the development of an individualized, supervised, and progressive therapeutic exercise program by selecting exercises that optimize the neuromuscular activity approaching the values of the healthy women group. The neuromuscular behavior in different scapular-focused exercises that have been proposed could be explored in shoulder pain dysfunctions, such as the scapular orientation exercises: the serratus punch, push-up plus, wall-slide, or towel wall-slide for the serratus anterior muscle; as well as side-lying external rotation or side-lying forward flexion, to promote neuromuscular coordination of the scapular stabilizer muscles [15,38,47,54,55,56,57,58]. The values obtained from the shoulder neuromuscular activity of the healthy women group could be used as a reference for the evaluation of the effectiveness of conservative treatments, such as physiotherapy [47].

Statistically significant differences were observed in some of the parameters of the neuromuscular activity of the different muscles and movements in women without pain following breast cancer treatment (G2) versus healthy women (G3). The perpetuation of these long-term modifications could induce a greater overload, activating the cycle that links nociception with neuromuscular alterations [24,27,47]. Therefore, physiotherapy treatment must be adapted to this situation to achieve good efficacy regardless of the presence or absence of pain [24,47].

One of the major limitations of this study is linked to both the sEMG procedure and the treatment of the electromyographic values. Frequently, sEMG values of maximal and/or submaximal isometric contractions are employed as a reference for data normalization [34]. This procedure is complicated to perform for those who suffer pain; therefore, in this study, it was normalized based on non-consensual criteria observed in previous studies performed in women after breast cancer treatment [5,8,20]. Among the studies reviewed, we found a high variability referring to the values from the sEMG, the kind of contractions demanded, the contraction performance speed, and the data quantification procedure. This complicated the comparison and interpretation with the found data from the diverse studies.

## 5. Conclusions

Women after breast cancer treatment show significant alterations in their shoulder neuromuscular activity. These alterations are of greater magnitude with the existence of peripheral persistent pain. Therefore, on the basis of these findings, conservative treatments, such as physiotherapy should develop a selective therapeutic exercise program that optimizes shoulder neuromuscular activity in women after breast cancer treatment.

## Figures and Tables

**Table 1:** Demographic, anthropometric, therapeutic, and clinical outcomes.

Outcomes	Group 1 (*N* = 30)	Group 2 (*N* = 30)	Group 3 (*N* = 30)	*p*
**Age (years, X (SD))**	56 (8)	54 (8)	55 (8)	0.788 *
**BMI (Kg/m^2^, X (SD))**	25.4 (5.1)	24.4 (5)	23.5 (4.2)	0.283 *
**Occupation**				
Remunerated job (*n* (%))	12 (40)	9 (30)	10 (33.3)	0.484 **
Retired (*n* (%))	9 (30)	15 (50)	10 (33.3)	
Disable (*n* (%))	3 (10)	0	2 (6.7)	
Jobless (*n* (%))	6 (20)	6 (20)	8 (26.7)	
**Education level**				
No studies (*n* (%))	3 (10)	7 (23)	1 (3.3)	0.156 **
Primary (*n* (%))	9 (30)	8 (26.7)	10 (33.3)	
Secondary (*n* (%))	6 (20)	10 (33.3)	9 (30)	
Bachelor (*n* (%))	8 (26.7)	4 (13.3)	10 (33.3)	
Superior grade (*n* (%))	3 (10)	1 (3.3)	0	
Master/doctorate (*n* (%))	1 (3.3)	0	0	
**Surgical procedure**				
Lumpectomy (*n* (%))	26 (86.7)	24 (80)	-	0.488 **
Quadrantectomy (*n* (%))	4 (13.3)	6 (20)		
**Axillary surgical**				
Lymphadenectomy (*n* (%))	29 (96.7)	29 (96.7)	-	-
SNLB (*n* (%))	1 (3.3)	1 (3.3)		
**Chemotherapy (*n* (%))**	30 (100)	30 (100)	-	-
**Radiotherapy (*n* (%))**	30 (100)	30 (100)	-	-
**Hormonal Therapy (*n* (%))**	25 (83.3)	23 (76.7)	-	0.784 **
**Axillary web syndrome after surgery (*n* (%))**	28 (93.3)	29 (96.7)	-	NS **
**Lymphocele (*n* (%))**	1 (3.3)	2 (6.7)	-	NS **
**MPS (*n* (%))**	30 (100)	-		
**VAS (cm, X (SD))**	7.20 (1.12)			
**Characteristics of pain**				
Predominant neuropathic pain (*n* (%))	25 (83.3)	-		
Mix (neuropathic & nociceptive pain) (*n* (%))	30 (100)			

* test ANOVA; ** Chi-squared test. X: mean; SD: standard deviation; N/n: number; BMI: body max index; SLNB: sentinel lymph node biopsy; MPS: myofascial pain syndrome; VAS: visual analogue scale; NS: no significance (*p* > 0.999).

**Table 2:** The onset of muscle activity. Abduction movement.

Mm	Group 1 X (SD)	Group 2 X (SD)	Group 3 X (SD)	*p* *	Group 1 vs. Group 2				Group 1 vs. Group 3				Group 2 vs. Group 3			
					Dif X	95% CI		*p* **	Dif X	95% CI		*p* **	Dif X	95% CI		*p* **
						LL	UL			LL	UL			LL	UL	
**UT**	**−1.51 (2.24)**	**−0.36 (0.33)**	**−0.41 (0.37)**	**0.001**	**1.15**	**0.31**	**1.98**	**0.004**	**1.10**	0.26	1.94	**0.005**	0.04	−0.79	0.88	NS ***
**LT**	−0.23 (0.2)	0.16 (0.69)	1.12 (0.09)	**<0.001**	0.40	0.13	0.66	**0.001**	1.35	1.09	1.62	**<0.001**	0.95	0.69	1.22	**<0.001**
**MD**	1.85 (0.86)	0.76 (0.65)	0.22 (0.15)	**<0.001**	1.09	0.69	1.49	**<0.001**	1.62	1.23	2.02	**<0.001**	0.53	0.13	0.93	**0.004**
**I**	1.01 (1.81)	0.90 (0.27)	−0.10 (1.13)	**0.001**	0.10	−0.67	0.89	NS ***	1.12	0.33	1.90	**0.002**	1.01	0.23	1.80	**0.006**
**SA**	2.08 (1.08)	1.15 (0.54)	1.09 (0.21)	**<0.001**	0.93	0.48	1.38	**<0.001**	0.99	0.54	1.44	**<0.001**	0.06	−0.38	0.50	NS ***

* ANOVA Test; ** Bonferroni test; *** *p* > 0.999. Mm (muscles); UT (upper trapezius); LT (lower trapezius), MD (middle deltoid), I (infraspinatus), SA (serratus anterior); X (mean); SD (standard deviation); Dif X (mean difference); 95% CI (95% confidence interval); LL (lower limit); UL (upper limit); NS (no significance); bold data = *p* < 0.005.

**Table 3:** Onset of muscle activity. Flexion movement.

Mm	Group 1 X (SD)	Group 2 X (SD)	Group 3 X (SD)	*p* *	Group 1 vs. Group 2				Group 1 vs. Group 3				Group 2 vs. Group 3			
					Dif X	95% CI		*p* **	Dif X	95% CI		*p* **	Dif X	95% CI		*p* **
						LL	UL			LL	UL			LL	UL	
**UT**	**−0.33 (0.16)**	**−0.14 (0.13)**	**−0.1 (0.14)**	**<0.001**	**0.18**	**0.09**	**0.28**	**<0.001**	**0.22**	0.13	0.32	**<0.001**	0.03	−0.13	0.05	0.924
**LT**	0.59 (0.29)	0.86 (0.3)	0.98 (0.21)	**<0.001**	0.26	0.09	0.43	**0.001**	0.39	0.21	0.56	**<0.001**	0.12	0.04	0.30	0.228
**AD**	0.99 (0.61)	0.24 (0.37)	0.04 (0.14)	**<0.001**	0.74	0.48	1.01	**<0.001**	0.95	0.68	1.22	**<0.001**	0.20	−0.05	0.47	0.178
**I**	0.03 (0.18)	0.19 (0.23)	0.3 (0.26)	**<0.001**	0.15	0.01	0.3	**0.028**	0.26	0.12	0.41	**<0.001**	0.11	0.03	0.25	0.204
**SA**	1.25 (0.38)	0.75 (0.36)	0.51 (0.3)	**<0.001**	0.50	0.27	0.72	**<0.001**	0.74	0.52	0.96	**<0.001**	0.24	0.02	0.46	**0.026**

* ANOVA Test. ** Bonferroni test. Mm (muscles); UT (upper trapezius); LT (lower trapezius), AD (anterior deltoid), I (infraspinatus), SA (serratus anterior); X (mean); SD (standard deviation); Dif X (mean difference); 95% CI (95% confidence interval); LL (lower limit); UL (upper limit); bold data = *p* < 0.05.

**Table 4:** Onset of muscle activity. External rotation movement.

Mm	Group 1 X (SD)	Group 2 X (SD)	Group 3 X (SD)	*p* *	Group 1 vs. Group 2				Group 1 vs. Group 3				Group 2 vs. Group 3			
					Dif X	95% CI		*p* **	Dif X	95% CI		*p* **	Dif X	95% CI		*p* **
						LL	UL			LL	UL			LL	UL	
**UT**	**−0.09 (0.18)**	**0.009 (0.29)**	**0.09 (0.14)**	**0.005**	**0.10**	**0.02**	**0.24**	**0.178**	**0.18**	0.05	0.32	**0.003**	0.08	0.05	0.31	0.453
**LT**	2.01 (0.74)	1.5 (0.46)	1.49 (0.36)	**0.001**	0.45	0.10	0.79	**0.006**	0.52	0.17	0.86	**0.001**	0.06	−0.27	0.41	NS ***
**PD**	0.82 (0.47)	0.67 (0.38)	0.46 (0.29)	**0.002**	0.15	−0.09	0.40	0.378	0.36	0.11	0.61	**0.002**	0.20	−0.03	0.45	0.129
**I**	0.29 (0.52)	0.11 (0.36)	−0.03 (0.08)	**0.003**	0.18	−0.05	0.41	0.177	0.33	0.10	0.56	**0.002**	0.15	−0.08	0.38	0.340
**SA**	1.68 (0.54)	1.29 (0.54)	1.04 (0.33)	**<0.001**	0.39	0.08	0.69	**0.006**	0.64	0.33	0.94	**<0.001**	0.24	−0.05	0.55	0.154

* ANOVA Test **; Bonferroni test ***; *p* > 0.999. Mm (muscles); UT (upper trapezius); LT (lower trapezius), PD (posterior deltoid), I (infraspinatus), SA (serratus anterior); X (mean); SD (standard deviation); Dif X (mean difference); 95% CI (95% confidence interval); UL (upper limit); LL (lower limit); NS (no significance); bold data: *p* < 0.05.

**Table 5:** The amplitude of muscle activity (RMS%). Abduction movement.

Mm	Group 1 X (SD)	Group 2 X (SD)	Group 3 X (SD)	*p* *	Group 1 vs. Group 2				Group 1 vs. Group 3				Group 2 vs. Group 3			
					Dif X	95% CI		*p* **	Dif X	95% CI		*p* **	Dif X	95% CI		*p* **
						LL	UL			LL	UL			LL	UL	
**UP**	**58.24 (10.31)**	**38.50 (9.18)**	**34.11(12.99)**	**<0.001**	**19.74**	**12.82**	**26.65**	**<0.001**	**24.13**	17.21	31.04	**<0.001**	4.39	−2.52	11.30	0.374
**LT**	14.72 (5.37)	15.94 (3.85)	19.64 (4.30)	**<0.001**	1.21	1.65	4.09	0.909	4.92	2.04	7.79	**<0.001**	3.70	0.83	6.57	**0.007**
**MD**	75.41 (10.19)	65.97 (9.89)	59.74 (8.86)	**<0.001**	9.44	3.35	15.54	**0.001**	15.67	9.58	21.77	**<0.001**	6.23	0.13	12.32	**0.043**
**I**	6.52 (2.63)	10.07 (3.01)	10.99 (3.27)	**<0.001**	3.55	1.67	5.43	**<0.001**	4.47	2.59	6.35	**<0.001**	0.92	−2.8	0.96	0.709
**SA**	4.75 (2.25)	13.77 (5.9)	22.63 (5.31)	**<0.001**	9.02	6.01	12.02	**<0.001**	17.87	14.87	20.87	**<0.001**	8.85	5.85	11.85	**<0.001**

* ANOVA Test; ** Bonferroni test. Mm (muscles); UT (upper trapezius); LT (lower trapezius), MD (middle deltoid), I (infraspinatus), SA (serratus anterior); X (mean); SD (standard deviation); Dif X (mean difference); 95% CI (95% confidence interval); LL (lower limit); UL (upper limit); bold data: *p* < 0.05.

**Table 6:** The amplitude of muscle activity (RMS%). Flexion movement.

Mm	Group 1 X (SD)	Group 2 X (SD)	Group 3 X (SD)	*p* *	Group 1 vs. Group 2				Group 1 vs. Group 3				Group 2 vs. Group 3			
					Dif X	95% CI		*p* **	Dif X	95% CI		*p* **	Dif X	95% CI		*p* **
						LL	UL			LL	UL			LL	UL	
**UT**	**37.09 (10.25)**	**20.95 (6.75)**	**17.38 (5.11)**	**<0.001**	**16.14**	**11.30**	**20.98**	**<0.001**	**19.71**	14.87	24.55	**<0.001**	3.57	−1.27	8.41	0.226
**LT**	11.21 (3.99)	15.24 (4.36)	22.89 (7.68)	**<0.001**	4.03	0.50	7.56	**0.020**	11.68	8.14	15.21	**<0.001**	7.64	4.11	11.17	**<0.001**
**AD**	80.79 (9.68)	65.43 (13.70)	61.90 (12.17)	**<0.001**	15.35	7.8	22.89	**<0.001**	18.89	11.34	26.43	**<0.001**	3.53	−4.008	11.08	0.767
**I**	11.17 (5.67)	22.6 (12.41)	46.88 (11.49)	**<0.001**	11.42	4.93	12.92	**<0.001**	35.70	29.21	42.2	**<0.001**	24.28	17.78	30.77	**<0.001**
**SA**	6.8 (3.54)	17.32 (7.7)	38.23 (11.04)	**<0.001**	10.45	5.39	15.52	**<0.001**	31.36	26.30	36.43	**<0.001**	20.91	15.84	25.97	**<0.001**

* ANOVA Test; ** Bonferroni test. Mm (muscles); UT (upper trapezius); LT (lower trapezius), AD (anterior deltoid), I (infraspinatus), SA (serratus anterior); X (mean); SD (standard deviation); Dif X (mean difference); 95% CI (95% confidence interval); LL (lower limit); UL (upper limit); bold data = *p* < 0.05.

**Table 7:** The amplitude of muscle activity (RMS%). External rotation movement.

Mm	Group 1 X (SD)	Group 2 X (SD)	Group 3 X (SD)	*p* *	Group 1 vs. Group 2				Group 1 vs. Group 3				Group 2 vs. Group 3			
					Dif X	95% CI		*p* **	Dif X	95 % CI		*p* **	Dif X	95% CI		*p* **
						LL	UL			LL	UL			LL	UL	
**UT**	**33.15 (7.89)**	**22.01 (8.6)**	**18.45 (7.89)**	**<0.001**	**11.14**	**6.01**	**16.26**	**<0.001**	**14.7**	9.57	19.82	**<0.001**	3.56	−1.56	8.68	0.281
**LT**	17.14 (3.71)	28.84 (12.39)	32.57 (12.1)	**<0.001**	11.7	5.25	18.15	**<0.001**	15.43	8.98	21.88	**<0.001**	3.73	−10.17	2.71	0.484
**PD**	22.39 (7.48)	42.81 (16.95)	48.5 (12.02)	**<0.001**	20.42	12.38	28.46	**<0.001**	26.11	18.07	34.14	**<0.001**	5.68	−13.72	2.35	0.263
**I**	20.91 (7.34)	50.20 (20.73)	72.55 (8.62)	**<0.001**	29.29	20.69	37.89	**<0.001**	51.64	43.02	60.24	**<0.001**	22.35	13.75	30.94	**<0.001**
**SA**	17.09 (6.60)	28.67 (10.75)	59.86 (10.92)	**<0.001**	11.58	5.5	17.65	**<0.001**	42.77	36.69	48.84	**<0.001**	31.19	25.11	37.26	**<0.001**

* ANOVA Test; ** Bonferroni test. Mm (muscles); UT (upper trapezius); LT (lower trapezius), PD (posterior deltoid), I (infraspinatus), SA (serratus anterior); X (mean); SD (standard deviation); Dif X (mean difference); 95% CI (95% confidence interval); LL (lower limit); UL (upper limit); bold data: *p* < 0.05.
